# A Wide-Angle and PON Fully Polarimetric Retrodirective Array at the X Band

**DOI:** 10.3390/mi15121418

**Published:** 2024-11-26

**Authors:** Shuangdi Zhao, Lei Chen, Jicheng Pan, Tianling Zhang

**Affiliations:** 1 Key Laboratory of Antennas and Microwave Technology, Xidian University, Xi’an 710071, China; leichen@mail.xidian.edu.cn (L.C.); tianlingzhang@126.com (T.Z.); 2 Guangzhou Institute of Technology, Xidian University, Xi’an 710071, China; jcpan0719@163.com

**Keywords:** retrodirective array, tri-polarized, PON, scattering matrix, multiport network, wide-angle

## Abstract

A new type of fully polarimetric retrodirective array (RDA) using a PON-type structure is proposed in this paper. The fully polarimetric property is the result of the proposed phase conjugation circuits, which perform phase conjugation processing on the x, y, and z polarization electric field components of the incident wave when combined with a tri-polarized antenna array. It enables the retrodirective array to receive and retransmit an arbitrary polarized incident wave. The measured results of the monostatic radar cross-section (RCS) show that the −5 dB beam width of the array was greater than 95° at 9.6 GHz for different polarized incident waves. Furthermore, the proposed RDA has better retrodirectivity performance on arbitrary polarized incident waves when using a wide-beam antenna, and if we further incorporate modulation and demodulation into the circuits, it has the potential to be applied to the wireless communications field.

## 1. Introduction

Adaptive antenna arrays are well-suited for many applications in both military and commercial communication systems [1]. The increase in demand for high link gain and high secrecy has direct impacts on the design issues of these systems. By properly adjusting the phase of the received signal at each element of the array, the beam direction and radiation pattern of the array can be steered towards a specified angular position. The retrodirective array, which automatically retransmits a beam of echoes in the direction of the incident wave without any prior knowledge of the source location, simplifies the complex and bulky adaptive array and eliminates the need for digital signal processing algorithms [2,3,4,5,6,7,8,9,10].

Conventionally, RDAs have been widely designed and investigated based on a single polarization element [11]. Although polarization characteristics have been previously studied in conjunction with RDAs, polarization duplexing has been utilized, where a set of orthogonal polarizations are used for receiving and retransmitting operations with high isolation [12]. However, they still operate for single linear polarized cases only and require the RDA to be pointing at the interrogator with a particular orientation to minimize the polarization mismatch loss. Some efforts have been made to overcome these limitations by utilizing a dual-polarized antenna array [13,14,15,16]. Nevertheless, the dual-polarized antenna cannot fully sense all arbitrary polarization incident waves, especially when the incident wave’s polarization is perpendicular to its polarization direction. In addition, owing to the non-omnidirectional radiation pattern of microstrip patch antennas, they are only proficient at receiving and retransmitting incident waves when they approach from the broadside direction and exhibit limited effectiveness when signals arrive from the end-fire direction.

To further improve the system performance, Yijue Wang et al. proposed a fully polarimetric retrodirective array based on the Van Atta array, which is formulated by connecting ports with the same polarization in pairs using a microstrip line with an integer multiples wavelength length of a tri-polarized antenna. The measured monostatic radar cross-section (RCS) results showed that, for transverse magnetic (TM) incident waves, the width of a −3 dB monostatic RCS beam is 132°. For transverse electric (TE) incident waves, it is only 52° [17].

However, the Van Atta array is not as flexible and highly isolated as the PON-type RDA in terms of system flexibility and transmit–receive isolation. Instead of pairing the antennas with respect to the symmetric plane of the array using equal electrical length interconnects, the PON-type RDA retrodirects signals by conjugating the received phase at each antenna element [18,19]. In addition, the PON-type RDA not only allows devices to be built on a conformal surface but also allows simpler integration of standard electronic devices, modulation, and amplification of the signals [20,21,22]. Although the PON-type technique has these advantages, it may face the problems of interference suppression and high LO frequency. However, it is obvious that it is still the most popular phase conjugation technique. The phase detection and PLL techniques avoid using a high LO frequency, with excellent conversion gain and efficiency, but this technique increases the complexity of the circuit. The digital retrodirective array has a flexible structure and is multifunctional, but the system is more expensive as it uses a digital circuit and is restricted to the performance of the digital element [23,24,25,26,27].

However, a few implementations can be found in the literature addressing the PON-type RDA with fully polarimetric properties. Therefore, a prototype fully polarimetric RDA is proposed. By performing phase conjugation on three polarization electric field components of incident waves separately and then connecting the circuit to the corresponding port of the tri-polarized antenna, the retrodirectivity of arbitrary polarized incident waves can be achieved. A prototype PON fully polarimetric RDA is designed and fabricated at the X-band. The results show that the measured −5 dB beam width was greater than 95° at the X-band for the arbitrary polarized wave. 

This study is organized as follows. In Section 2, the retrodirectivity of a fully polarimetric retrodirective array is analyzed by conducting modular analysis. The design of the fully polarimetric RDA is provided in Section 3. Afterwards, experimental results are presented in Section 4 to validate the retrodirective property of the proposed fully polarimetric RDA. Finally, the conclusions are given in Section 5.

## 2. Analysis of the Retrodirectivity of the Fully Polarimetric Retrodirective Array

### 2.1. Fully Polarimetric Phase Conjugation Principle

As retrodirectivity is the result of the phase conjugation of the pilot signal received by each element of the array, the theory of fully polarimetric phase conjugation is studied and demonstrated based on the principle of generalized phase conjugation. Set the time factor as $e^{j\omega t}$, and the simple expression of the incident plane wave ${\vec{E}}_{in}$ propagating in the $\hat{k}$ direction is

(1) E⇀inr⇀,t=ReE⇀r⇀e−jk⇀⋅r⇀ejωt,

where $\vec{E}\left(\vec{r}\right)$ represents the complex amplitude and $\vec{k}=k\hat{k}$ represents the wave vector. The phase conjugation wave retransmitted by the retrodirective array system is obtained, which is called a generalized phase conjugation wave as shown in Equation (2):

(2) E⇀cr⇀,t=ReρE⇀∗r⇀ejk⇀⋅r⇀ejωct,

where ρ is an arbitrary real constant introduced in the transmission link and $\omega_{c}$ is the angular frequency of the phase conjugation wave. It shows that the propagation direction of phase-conjugated waves is opposite to ${\vec{E}}_{in}\left(\vec{r},t\right)$ (i.e., $-{\vec{k}}_{in}$), its complex amplitude is the conjugation of $\vec{E}\left(\vec{r}\right)$, and the spatial distribution of its phase is the same as $\vec{E}\left(\vec{r}\right)$.

Based on the generalized phase conjugation of a single linearly polarized incident wave, the fully polarimetric phase conjugation principle can be derived. For a monochromatic electromagnetic wave of arbitrary polarization, its polarization is decomposed into three orthogonal components, and each coordinate component is studied separately. Taking a Cartesian coordinate system as an example, the incident plane wave in the direction of ${\vec{k}}_{in}$ can be represented as:

(3) E⇀inr⇀,t=ReaE⇀xr⇀ej(ωxt−k⇀x⋅r⇀)+RebE⇀yr⇀ej(ωyt−k⇀y⋅r⇀)+RecE⇀zr⇀ej(ωzt−k⇀z⋅r⇀),

where ${\vec{E}}_{x}\left(\vec{r}\right)$, ${\vec{E}}_{y}\left(\vec{r}\right)$, and ${\vec{E}}_{z}\left(\vec{r}\right)$ represent the complex amplitudes of three electric field components of the incident wave, respectively, and the corresponding wave vectors are ${\vec{k}}_{x}=k{\hat{k}}_{x}$, ${\vec{k}}_{y}=k{\hat{k}}_{y}$, and ${\vec{k}}_{z}=k{\hat{k}}_{z}$.

The phase conjugation wave can be defined as

(4) E→cr→,t=Reρ1E→x∗r→ej(ωxt+k→x⋅r→+α1)+Reρ2E→y∗r→ej(ωyt+k→y⋅r→+α2)+Reρ3E→z∗r→ej(ωzt+k→z⋅r→+α3),

where $\rho_{1}$, $\rho_{2}$, $\rho_{3}$ and $\alpha_{1}$, $\alpha_{2}$, $\alpha_{3}$, respectively, represent additional amplitude and phase factors introduced by the circuits. For an arbitrary polarized incident wave, the amplitude and phase of different polarization components should meet Equations (5) and (6).

(5) $$\rho_{1}=ha,\rho_{2}=hb,\rho_{3}=hc,$$

and

(6) $$\alpha_{1}=\alpha_{2}=\alpha_{3},$$

where h is a real constant. When the generalized phase conjugation condition is satisfied in all electric field polarization components, then the transmission direction of phase conjugation wave ${\vec{E}}_{c}\left(\vec{r},t\right)$ is opposite to ${\vec{E}}_{in}\left(\vec{r},t\right)$ (i.e., $-{\vec{k}}_{in}$). If the key parameters mentioned above can be met perfectly in the design, fully polarimetric retrodirectivity will be realized.

### 2.2. The Model of a Fully Polarimetric Phase Conjugation System

As shown in [28], it is possible to cast the retrodirective array as a multiport *S*-parameter problem, and in this way systematically include the effects of multiple coupling paths between elements and reflection caused by a mismatch between the antenna and feeding system in the system. Assuming that there are *k* + 1 sources simultaneously transmitting signals to the retrodirective array and waiting for responses, the retrodirective array has *N* − *k* elements, where $\left[a_{0},a_{1},\cdots a_{N}\right]$ and $\left[b_{0},b_{1},\ldots b_{N}\right]$, respectively, represent the transmitting and receiving signals. The system can be represented by means of the multiport *S*-parameter scattering matrix, which can be divided into four parts.

(7) $$S=\left[\begin{array}{cc} S_{A} & S_{B}\\ S_{C} & S_{D} \end{array}\right],$$

Let the mutual coupling between *k* + 1 signal sources be represented as

(8) $$S_{A}=\left[\begin{array}{cccc} S_{00} & S_{01} & \cdots & S_{0k}\\ S_{10} & S_{11} & \cdots & S_{1k}\\ \vdots & \vdots & \ddots & \vdots \\ S_{k0} & S_{k1} & \cdots & S_{kk} \end{array}\right],$$

Let the process of transmission from the signal sources to the elements of the retrodirective array be represented as

(9) $$S_{B}=\left[\begin{array}{ccc} S_{0k+1} & \cdots & S_{0N}\\ S_{1k+1} & \cdots & S_{1N}\\ \vdots & \ddots & \vdots \\ S_{kk+1} & \cdots & S_{kN} \end{array}\right],$$

Let the process of transmission from the retrodirective array to the signal sources be represented as

(10) $$S_{C}=\left[\begin{array}{cccc} S_{(k+1)0} & S_{(k+1)1} & \cdots & S_{(k+1)k}\\ \vdots & \vdots & \ddots & \vdots \\ S_{N0} & S_{N1} & \cdots & S_{Nk} \end{array}\right],$$

Let the mutual coupling and reflection coefficients between the elements of the retrodirective array be represented as

(11) $$S_{D}=\left[\begin{array}{ccc} S_{\left(k+1)(k+1\right)} & \cdots & S_{(k+1)N}\\ \vdots & \ddots & \vdots \\ S_{N(k+1)} & \cdots & S_{NN} \end{array}\right],$$

However, the existing study on the multiport scattering matrix theory for the retrodirective array system has two significant limitations. Firstly, it assumed an ideal feeding network structure that is perfectly matched and decoupled. Secondly, it did not take the polarization characteristics of the incident wave into account. However, in practical applications, challenges such as mutual coupling between signal sources, mismatches between the feeding network structure and antennas, and air propagation loss impact the accuracy of theoretical predictions. Therefore, we address these issues by deducing a more practical analysis process for the fully polarimetric retrodirective array system.

**Figure 1:**
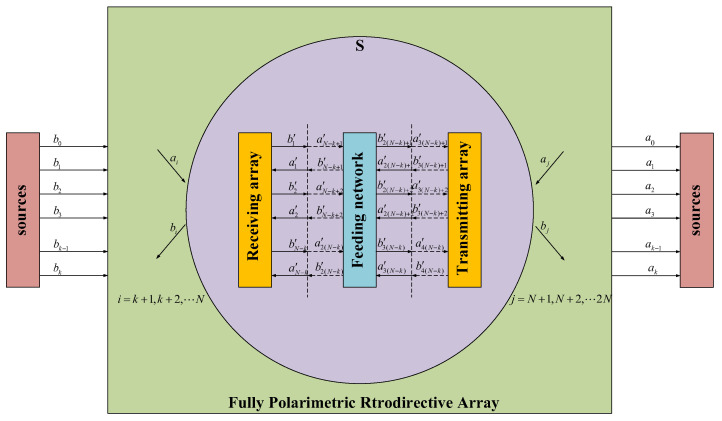
The model of the fully Polarimetric retrodirective array system.

The proposed model is divided into a transmitting array, a feeding network, and a receiving array. As shown in Figure 1, we set some settings as follows:

1. $\left[b_{0},b_{1}\cdots b_{k}\right]$, $\left[a_{0},a_{1}\cdots a_{k}\right]$, respectively, represent the signal emitted by a single signal source and the distribution of signals received by sources in the system;
2. A spherical *S* is selected that completely includes the retrodirective array reception and radiation space. $\left(a_{i},b_{i}\right)(i=k+1,k+2,\cdots N)$, $\left(a_{i},b_{i}\right)(i=N+1,N+2,\cdots 2N)$, respectively, represent the complex amplitudes of the *i*-th incident spherical wave $e_{i}^{in}$ and the *i*-th outgoing spherical wave $e_{i}^{out}$ in the function expansion of the receiving field and the reradiation field;
3. It is assumed that there are *N* − *k* elements in the receiving array that can be seen as *N* − *k* ports, let these be represented as $S_{D}$, and let the transmitting array be represented as $S_{D}^{\prime }$ in the same way. In the single-mode transmission area, where elements of the transmitting and receiving arrays are connected to the feeding network, a cross-section is taken as the port reference plane of that element to form another reference plane in the model. We assumed that the ports corresponding to *N* − *k* elements of the receiving array on this reference plane are ports No. (*N* − *k* + 1) to No. 2 (*N* − *k*), and the ports corresponding to *N* − *k* elements of the transmitting array on this reference plane are ports No. 2 (*N* − *k*) + 1 to No. 3 (*N* − *k*);
4. The feeding network becomes a 2(*N* − *k*)-port network, with an *S*-parameter matrix of $S^{p}$, where $a_{i}^{\prime }\left(i=1\cdots 4(N-k)\right)$ and $b_{i}^{\prime }\left(i=1\cdots 4(N-k)\right)$, respectively, represent the input and output of the feeding network.

To make the expression concise, we make

(12) $$\begin{array}{c} A_{1}={\left[a_{1}^{\prime },a_{2}^{\prime },\cdots a_{N-k}^{\prime }\right]}^{T}\\ A_{2}={\left[a_{N-k+1}^{\prime },a_{N-k+2}^{\prime },\cdots a_{2(N-k)}^{\prime }\right]}^{T}\\ A_{3}={\left[a_{2(N-k)+1}^{\prime },a_{2(N-k)+2}^{\prime },\cdots a_{3(N-k)}^{\prime }\right]}^{T}\\ A_{4}={\left[a_{3(N-k)+1}^{\prime },a_{3(N-k)+2}^{\prime },\cdots a_{4(N-k)}^{\prime }\right]}^{T} \end{array},$$

(13) $$\begin{array}{c} B_{1}={\left[b_{1}^{\prime },b_{2}^{\prime },\cdots b_{N-k}^{\prime }\right]}^{T}\\ B_{2}={\left[b_{N-k+1}^{\prime },b_{N-k+2}^{\prime },\cdots b_{2(N-k)}^{\prime }\right]}^{T}\\ B_{3}={\left[b_{2(N-k)+1}^{\prime },b_{2(N-k)+2}^{\prime },\cdots b_{3(N-k)}^{\prime }\right]}^{T}\\ B_{4}={\left[b_{3(N-k)+1}^{\prime },b_{3(N-k)+2}^{\prime },\cdots b_{4(N-k)}^{\prime }\right]}^{T} \end{array},$$

The field in the outer space of *S* can be expanded using the spherical wave vector wave function as the basis:

(14) $$E_{r}=\sum_{i=k+1}^{N}\left(a_{i}e_{i}^{in}+b_{i}e_{i}^{out}\right),$$

(15) $$E_{t}=\sum_{i=N+1}^{2N}\left(a_{i}e_{i}^{in}+b_{i}e_{i}^{out}\right),$$

where $E_{r}$ and $E_{t}$, respectively, represent the receiving field and reradiation field of the retrodirective array. To make the expression concise, we make

(16) $$\begin{array}{c} A_{0}={\left[a_{k+1},a_{k+2},\cdots a_{N}\right]}^{T}\\ B_{0}={\left[b_{k+1},b_{k+2},\cdots b_{N}\right]}^{T}\\ A_{5}={\left[a_{N+1},a_{N+2},\cdots a_{2N}\right]}^{T}\\ B_{5}={\left[b_{N+1},b_{N+2},\cdots b_{2N}\right]}^{T} \end{array},$$

During the stage of receiving signals from the signal sources, we can make $B_{0}=0$. During the stage of radiating signals from the retrodirective array, we can make $A_{5}=0$.

### 2.3. Analysis of Retrodirectivity for a Fully Polarimetric Retrodirective Array

#### 2.3.1. The Receiving Course of the RDA

The signal emitted by a single source is expanded using a spherical wave vector function as the basis in the Cartesian coordinate system. The spherical wave expansion formula of the field can be expressed as

(17) $$E_{ti}=c_{ix}e^{j\psi_{ix}}{\hat{e}}_{x}+c_{iy}e^{j\psi_{iy}}{\hat{e}}_{y}+c_{iz}e^{j\psi_{iz}}{\hat{e}}_{z},$$

It can also be expressed in the vector form as

(18) $$E_{ti}=\left[\begin{array}{ccc} c_{ix}e^{j\psi_{ix}} & c_{iy}e^{j\psi_{iy}} & c_{iz}e^{j\psi_{iz}} \end{array}\right],$$

where $c_{ix}$, $c_{iy}$, $c_{iz}$ and $\psi_{ix}$, $\psi_{iy}$, $\psi_{iz}$ represent the amplitude and phase of the incident wave from the *i*-th signal source in the directions of the x-, y-, and z-axis, respectively.

Therefore, the signal emitted by a single signal source can be represented as

(19) $$b_{i}=\left[\begin{array}{ccc} {\dot{c}}_{ix} & {\dot{c}}_{iy} & {\dot{c}}_{iz} \end{array}\right],$$

where ${\dot{c}}_{ix}$, ${\dot{c}}_{iy}$, ${\dot{c}}_{iz}$ represent the complex amplitude of the incident wave of the *i*-th signal source in the directions of the x-, y-, and z-axis, respectively.

Without considering multipath propagation, the process of signal transmission from the sources to each element of the retrodirective array in the system can be expressed as follows:

(20) $$\left[b_{0},b_{1}\cdots b_{k}\right]S_{B}=\left[b_{0},b_{1}\cdots b_{k}\right]\left[\begin{array}{ccc} S_{0k+1} & \cdots & S_{0N}\\ S_{1k+1} & \cdots & S_{1N}\\ \vdots & \ddots & \vdots \\ S_{kk+1} & \cdots & S_{kN} \end{array}\right],$$

where the elements $S_{ij}\left(i=0,1,\cdots k;j=k+1,k+2,\cdots N\right)$ in the matrix $S_{B}$ can be represented as

(21) $$S_{ij}=\left[\begin{array}{ccc} S_{ij}^{x,x} & S_{ij}^{x,y} & S_{ij}^{x,z}\\ S_{ij}^{y,x} & S_{ij}^{y,y} & S_{ij}^{y,z}\\ S_{ij}^{z,x} & S_{ij}^{z,y} & S_{ij}^{z,z} \end{array}\right],$$

where $S_{ij}^{m,n}$ represents the complex amplitude and phase change from n-polarization to m-polarization when the transmission signal from the *j*-th source is transmitted to the *i*-th element of the retrodirective array.

Then, the signal distribution of each port in the retrodirective array of the system is

(22) $$\left[a_{k+1},a_{k+2},\cdots a_{N}\right],$$

where

(23) $$a_{n}=\sum_{i=0}^{k}b_{i}S_{in};(k+1\leq n\leq N),$$

#### 2.3.2. The Retransmitting Course of the RDA

According to the established model, the characteristics of the receiving array can be represented by the *S*-parameter equation as follows:

(24) $$\left[\begin{array}{c} B_{0}\\ B_{1} \end{array}\right]=\left[\begin{array}{cc} S_{11}^{A} & S_{12}^{A}\\ S_{21}^{A} & S_{22}^{A} \end{array}\right]\left[\begin{array}{c} A_{0}\\ A_{1} \end{array}\right],$$

The *S*-parameter equation of the feeding network can be expressed as follows:

(25) $$\left[\begin{array}{c} B_{2}\\ B_{3} \end{array}\right]=\left[\begin{array}{cc} S_{11}^{p} & S_{12}^{p}\\ S_{21}^{p} & S_{22}^{p} \end{array}\right]\left[\begin{array}{c} A_{2}\\ A_{3} \end{array}\right],$$

We had

(26) $$\begin{array}{l} A_{2}=B_{1},B_{2}=A_{1}\\ A_{4}=B_{3},B_{4}=A_{3} \end{array},$$

and

(27) $$S_{22}^{A}=S_{D},S_{11}^{B}=S_{D}^{\prime },$$

and

(28) $$\begin{array}{l} A_{2}=B_{1},B_{2}=A_{1}\\ A_{4}=B_{3},B_{4}=A_{3} \end{array},$$

Assuming the *S*-parameter matrix of the entire network is [S]

(29) $$\left[S\right]=\left[\begin{array}{cc} S_{11} & S_{12}\\ S_{21} & S_{22} \end{array}\right],$$

then

(30) $$\left[\begin{array}{c} B_{0}\\ B_{5} \end{array}\right]=\left[S\right]\left[\begin{array}{c} A_{0}\\ A_{5} \end{array}\right]=\left[\begin{array}{cc} S_{11} & S_{12}\\ S_{21} & S_{22} \end{array}\right]\left[\begin{array}{c} A_{0}\\ A_{5} \end{array}\right],$$

where we can calculate that the amplitude vector of the radiation wave from the retrodirective array is as follows

(31) $$B_{5}=S_{21}A_{0}+S_{22}A_{5}=S_{21}A_{0},$$

Let $S_{C}=\left[\begin{array}{cc} S_{11}^{C} & S_{12}^{C}\\ S_{21}^{C} & S_{22}^{C} \end{array}\right]$ be the *S*-parameter matrix after cascading $S_{A}$ and $S_{B}$, and we can calculate the result by using the generalized cascading network *S*-parameter formula

(32) $$S_{21}=S_{21}^{B}{\left\{I-S_{11}^{B}S_{22}^{C}\right\}}^{-1}S_{21}^{C},$$

Similarly, the process of signals radiating from the retrodirective array to sources in the system can be expressed as follows:

(33) $$B_{5}^{T}S_{C}=\left[b_{N+1},b_{N+2},\cdots b_{2N}\right]\left[\begin{array}{cccc} S_{(k+1)0} & S_{(k+1)1} & \cdots & S_{(k+1)k}\\ \vdots & \vdots & \ddots & \vdots \\ S_{N0} & S_{N1} & \cdots & S_{Nk} \end{array}\right],$$

where the vector element $S_{ij}\left(i=k+1,k+2,\cdots N;j=0,1,\cdots k\right)$ of matrix $S_{C}$ can be represented as

(34) $$S_{ij}=\left[\begin{array}{ccc} S_{ij}^{x,x} & S_{ij}^{x,y} & S_{ij}^{x,z}\\ S_{ij}^{y,x} & S_{ij}^{y,y} & S_{ij}^{y,z}\\ S_{ij}^{z,x} & S_{ij}^{z,y} & S_{ij}^{z,z} \end{array}\right],$$

where $S_{ij}^{m,n}$ represents the complex amplitude phase change from n-polarization to m-polarization of the reradiation signal transmitted from the *j*-th elements of the retrodirective array to the *i*-th source.

Finally, the distribution of signals received by sources in the system is

(35) $$\left[a_{0},a_{1}\cdots a_{k}\right]=B_{5}^{T}S_{C}={\left(S_{21}A_{0}\right)}^{T}S_{C},$$

The example of arbitrary polarimetric electromagnetic waves from multiple signal sources is provided to verify the effectiveness of the proposed method. It assumes that there is no mutual coupling and mismatching between signal sources and the elements of the retrodirective array. 

Taking three signal sources as examples, the transmission signals can be represented as

(36) $$\left[b_{0},b_{1},b_{2}\right],$$

where $b_{i}=\left[\begin{array}{ccc} {\dot{c}}_{ix} & {\dot{c}}_{iy} & {\dot{c}}_{iz} \end{array}\right]\left(i=1,2,3\right)$ and ${\dot{c}}_{ix}$, ${\dot{c}}_{iy}$, ${\dot{c}}_{iz}$, respectively, represent the complex amplitude of the incident wave from the *i*-th signal source in the directions of the x-, y-, and z-axis.

For simplicity, we ignore the mutual influence between signal sources and the mutual coupling between the elements of the retrodirective array, where $S_{A}$ and $S_{B}$ are both zero, and the multiport scattering matrix is simplified as

(37) $$S=\left[\begin{array}{cc} 0 & S_{B}\\ S_{C} & 0 \end{array}\right],$$

Without considering multipath propagation, the process of signal transmission from the signal sources to each element of the retrodirective array in the system can be expressed in the form of vector and matrix multiplication

(38) $$\left[b_{0},b_{1},b_{2}\right]S_{B}=\left[b_{0},b_{1},b_{2}\right]\left[\begin{array}{ccc} S_{0k+1} & \cdots & S_{0N}\\ S_{1k+1} & \ddots & S_{1N}\\ S_{2k+1} & \cdots & S_{2N} \end{array}\right],$$

The result of Equation (38) is Equation (39), which represents the signal distribution of each element port in the retrodirective array of the system.

(39) $$\left[a_{k+1},a_{k+2},\cdots a_{N}\right],$$

where

(40) $$a_{n}=\sum_{i=0}^{k}b_{i}S_{in}\;(k+1\leq n\leq N),$$

Therefore, the signal vector received by the retrodirective array can be expressed as

(41) $$A_{0}={\left[a_{k+1},a_{k+2},\cdots a_{N}\right]}^{T}={\left[\sum_{i=0}^{k}b_{i}S_{ik+1},\sum_{i=0}^{k}b_{i}S_{ik+2},\cdots \sum_{i=0}^{k}b_{i}S_{iN}\right]}^{T},$$

Assuming that the transmitting and receiving arrays are perfectly matched with the feeding network, the reradiated wave can be expressed as

(42) $$B_{5}=S_{21}^{B}S_{21}^{p}S_{21}^{A}A_{0},$$

where $S_{21}^{A}$ and $S_{21}^{B}$, respectively, represent the gain of the transmitting and receiving arrays, and according to the characteristics of the phase conjugation circuit, $∠\phi (S_{21}^{p})=-2∠\phi (A_{0})$, we can obtain

(43) $$∠\phi \left(B_{5}\right)=-∠\phi \left(A_{0}\right),$$

This result satisfies the phase conjugation condition. It is shown in Table 1 that if the retrodirectivity of the fully polarimetric retrodirective array is estimated and tested according to the proposed method and then error analysis is carried out, the results are closer to real-life applications, thus providing good theoretical guidance for the analysis of retrodirectivity in the fully polarimetric retrodirective array. 

## 3. A Fully Polarimetric Retrodirective Array

**Figure 2:**
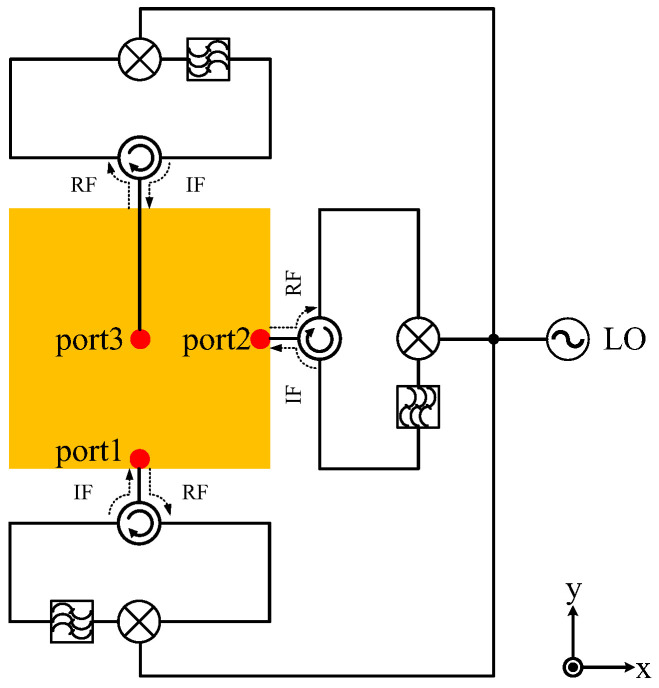
Schematic of an element in the fully polarimetric RDA based on a PON-type structure.

The schematic of an element in the proposed retrodirective antenna is shown in Figure 2. It consists of a tri-polarized antenna and fully polarimetric phase conjugation circuits comprising three phase conjugation circuits, each of which contains a circulator, mixer, and filter and is supplied by the same local oscillator. In practical design, flexibility can be incorporated, such as employing two separate receiving and transmitting antennas instead of a circulator, integrating amplifiers into the phase conjugate circuit to achieve higher gains, or introducing modulation/demodulation for communication applications, etc.

If we assume an incoming signal $V_{RF}\cos\left(\omega t+\phi_{RF}\right)$ is applied to a mixer with a local oscillator (LO) running at $V_{LO}\cos\left(2\omega t+\phi_{LO}\right)$, then the desired output of the mixer is $V_{IF}\cos\left(\omega t+\phi_{LO}-\phi_{RF}\right)$ and the phase of the incoming signal is automatically conjugated. If each phase conjugation circuit is well-designed and meets Equations (5) and (6), the fully polarimetric retrodirective array is made based on a PON structure. It is not as strict as a linearly polarized RDA, whose placement posture requires complete alignment, and allows for changing postures in application. In addition, conformal array forms can be adopted to expand its applications.

### 3.1. Wide-Beam Tri-Polarized Antenna Design

**Figure 3:**
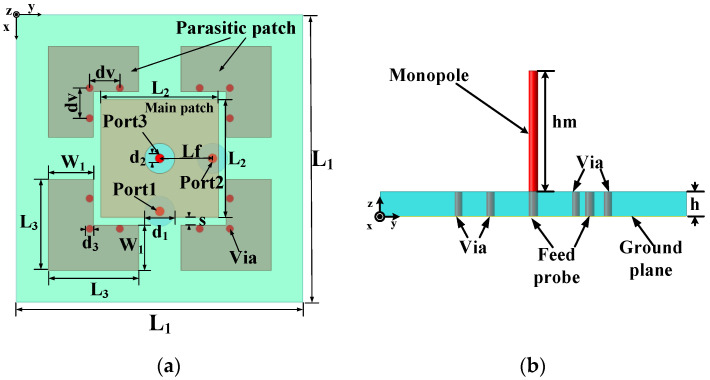
Structure of the tri-polarized antenna. (**a**) Top view. (**b**) Side view. (L1 = 19 mm, L2 = 7.8 mm, L3 = 6 mm, d1 = 2 mm, d2 = 0.51 mm, d3 = 0.5 mm, dv = 2 mm, s = 0.5 mm, W1 = 2.5 mm, h = 1.5 mm, hm = 7.5 mm, Lf = 3.5 mm).

In order to obtain a wide range of responding angles, broad-beam antennas can be used in the receiving and transmitting ends of the transponder. The structure of the tri-polarized antenna is shown in Figure 3 [29]. Port 1 and port 2 are the excitation ports for x-axis and y-axis polarization of the microstrip patch, respectively. Port 3 is the excitation port of the monopole, and the antenna array uses a F4B substrate ($\varepsilon_{r}$ = 2.2 and tanδ = 0.001) with a thickness of 1.5 mm. The working bandwidth of the tri-polarized antenna array is 160 MHz from 9.52 GHz to 9.68 GHz. 

The measured beam width in the E/H-plane is 150°/82° and 174°/70° when port 1 and port 2 are excited, respectively. When port 3 is excited, the antenna pattern is unipolar. The results meet the requirements of a wide-beam tri-polarized antenna array design.

### 3.2. Fully Polarimetric Phase Conjugation Circuit Design

**Figure 4:**
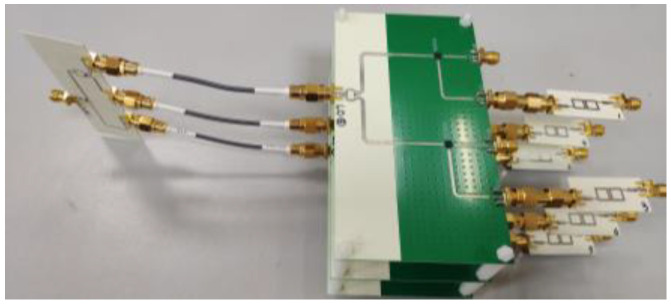
Photograph of the fully polarimetric phase conjugate circuit.

The receiving and retransmitting elements are designed separately to replace the circulator, which makes the structure of the phase conjugation circuits much simpler. The photograph of the fully polarimetric phase conjugate circuit is shown in Figure 4. The phase conjugation circuits comprise six channels, each of which includes a 9.6 GHz bandpass filter and a MAMX-011036 Double-Balanced Mixer (Mansfield, TX, USA). Finally, they all connect to a local oscillator at 19.2 GHz through a one-to-two Wilkinson power divider and a one-to-three Wilkinson power divider. The leakage of the RF-IF and LO-IF mixers was measured to be 9 dB and 40 dB, respectively, and the frequency conversion loss was measured to be 10 dB.

**Figure 5:**
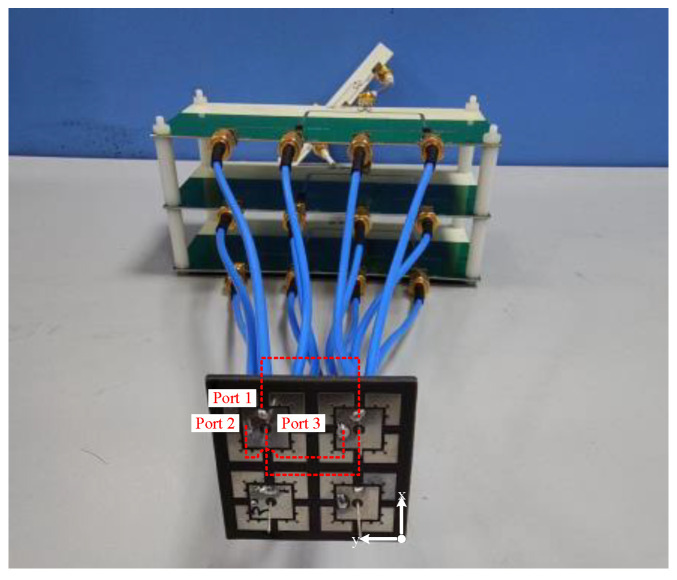
Schematic of an element in the fully polarimetric RDA based on a PON-type structure.

Two 2-element liner arrays are designed in the prototype for receiving and retransmitting, as shown in Figure 5.

## 4. Performance of the RDA

**Figure 6:**
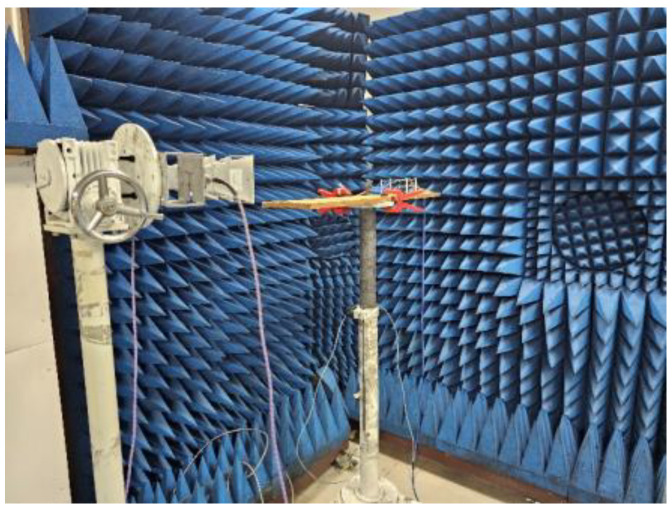
Measurement in the anechoic chamber.

In the test setup, a small frequency offset between receiving and retransmitting the signal was introduced to improve isolation discrimination and ensure minimum interference at the monostatic sensor. As shown in Figure 6, two horn antennas are placed in the same position, with one horn serving as the transmission source and the other horn receiving the backtracking wave. The RDA operated at 9.59 GHz for receiving and 9.61 GHz for retransmitting and the local oscillator operated at 19.2 GHz. The proposed RDA can work for any polarized wave at the working bandwidth of the tri-polarized antenna array (9.52 GHz to 9.68 GHz); however, the array was tested experimentally only for linear polarization due to only linearly polarized horns being available.

**Figure 7:**
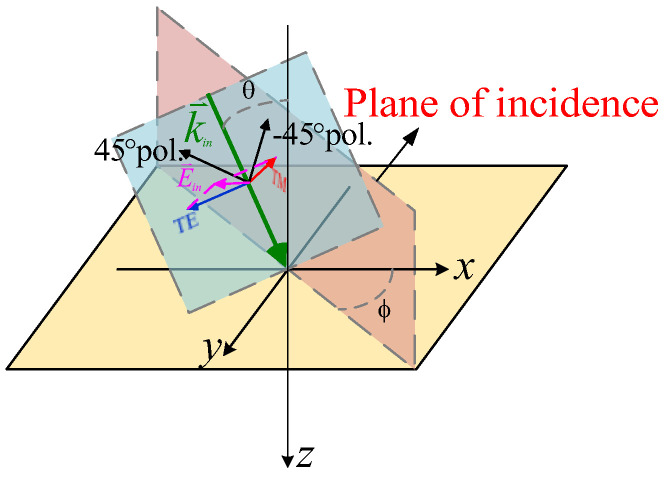
Incident plane wave with different polarizations.

In order to analyze the fully polarimetric retrodirectivity, the monostatic RCS test was implemented with different polarized incident waves. As shown in Figure 7, when the polarization direction of the incident wave is perpendicular to or inside the incident plane (yoz plane), it is defined as a TE or TM incident plane wave, respectively. When the polarization direction of the incident wave forms an angle of 45° or −45° with respect to the incident surface, it is marked as a 45°- or −45°-polarized plane wave, respectively.

**Figure 8:**
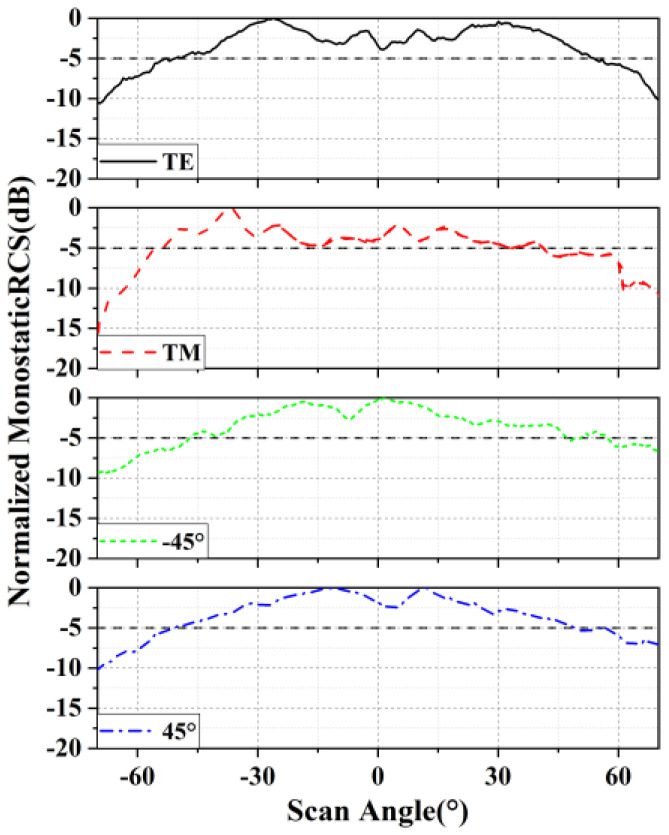
Measured (@9.61 GHz) monostatic RCS.

As can be seen from Figure 8, the width of the measured −5 dB beam is 106.56° (−52.74°~53.82°) for the TE wave, 97.92° (−55.54°~41.66°) for the TM wave, 101.72° (−52.34°~49.38°) for the 45°-polarized plane wave, and 104.04° (−57.06°~46.98°) for the −45°-polarized plane wave. It can be noted that the proposed fully polarimetric RDA achieved good retrodirective performance for different polarized waves. In addition, the working bandwidth of the RDA is 160 MHz from 9.52 GHz to 9.68 GHz, although we only provided results for 9.61 GHz here. Additionally, a comparison of our proposed RDA with some of the latest and most classic designs is summarized in Table 2. It can be observed that compared with other RDAs, the proposed RDA provides overall improved performance and an effective retrodirective performance with arbitrary polarized TM and TE incident waves, with an impressive monostatic RCS angle range.

## 5. Conclusions

A fully polarimetric retrodirective array is proposed, which combines fully polarimetric phase conjugation circuits with a tri-polarized receiving and transmitting antenna array. Then, the feasibility of retrodirecting arbitrary polarized incident waves is verified by using the generalized phase conjugation principle in theory. Finally, a monostatic RCS measurement is conducted while changing the polarization of the incident wave. The results show that the measured −5 dB beam width of monostatic RCS is greater than 95° at the X band for different polarized incident waves. The results have verified that the proposed fully polarimetric RDA can receive and retransmit arbitrary incident waves. Compared with the Van Atta array, the proposed RDA has the advantages of excellent performance and flexible and diverse application scenarios. It can not only retrodirect arbitrary incident waves but also broaden the range of retrodirective angles, thereby expanding the applications of the RDA in military and civilian mobile wireless systems.

## Figures and Tables

**Table 1:** The advantages and disadvantages of the proposed method.

Method	Advantages	Disadvantages
This work	(1) more practical: includes the mismatches between the feeding network structure and antennas, air propagation loss, and mutual coupling between signal sources; (2) can be used for analyzing a fully polarimetric retrodirective array as it takes the polarization characteristics of the incident wave into account.	The calculation process is a bit complicated and not suitable for situations where only a rough performance of the RDA estimate is needed.

**Table 2:** Comparison of the proposed RDA’s performances with previous studies.

Research	Center Operating Frequency	Polarization	Unit Number	Monostatic RCS Beam Width
[30]	0.99 GHz (Rx)/0.97 GHz (Tx)	S-LP	2	50° (−3 dB)
[31]	7.1 GHz (Rx)/8.4 GHz (Tx)	D-LP	4	90° (Not mentioned)
[32]	2.2 GHz (Rx)/2.4 GHz (Tx)	S-CP	5	80° (Not mentioned)
[33]	2.4 GHz (Rx)/2.5 GHz (Tx)	S-CP	16	60° (−5 dB)
[17]	5.6 GHz	Tri-P	4	52° (−3 dB)
This work	9.6 GHz	Tri-P	4	95° (−5 dB)

S-LP = Single Linear Polarization; D-LP = Dual Linear polarization; S-CP = Single Circular Polarization; Tri-P = Tri-polarization.

## Data Availability

The original contributions presented in the study are included in the article, further inquiries can be directed to the corresponding author.
